# Modelling of Amperometric Biosensor Used for Synergistic Substrates Determination

**DOI:** 10.3390/s120404897

**Published:** 2012-04-16

**Authors:** Dainius Simelevicius, Romas Baronas, Juozas Kulys

**Affiliations:** 1 Faculty of Mathematics and Informatics, Vilnius University, Naugarduko 24, LT-03225 Vilnius, Lithuania; E-Mail: romas.baronas@mif.vu.lt; 2 Institute of Biochemistry, Vilnius University, Mokslininku 12, LT-08662 Vilnius, Lithuania; E-Mail: juozas.kulys@bchi.vu.lt

**Keywords:** modelling, simulation, reaction-diffusion, biosensor, synergistic substrates

## Abstract

In this paper the operation of an amperometric biosensor producing a chemically amplified signal is modelled numerically. The chemical amplification is achieved by using synergistic substrates. The model is based on non-stationary reaction-diffusion equations. The model involves three layers (compartments): a layer of enzyme solution entrapped on the electrode surface, a dialysis membrane covering the enzyme layer and an outer diffusion layer which is modelled by the Nernst approach. The equation system is solved numerically by using the finite difference technique. The biosensor response and sensitivity are investigated by altering the model parameters influencing the enzyme kinetics as well as the mass transport by diffusion. The biosensor action was analyzed with a special emphasis to the effect of the chemical amplification. The simulation results qualitatively explain and confirm the experimentally observed effect of the synergistic substrates conversion on the biosensor response.

## 1. Introduction

A biosensor is an electronic measuring device designed for measurement of the concentration of some specific substance (analyte) in a solution. The device specificity for a particular substance is achieved by some biological material, usually an enzyme [1–3]. An amperometric biosensor assesses the concentration of the analyte through the measurement of the current on a working electrode [4,5]. Biosensors are widely used in various applications that require fast quantitative analysis [6–10].

Oxidases are the kind of enzymes commonly used in biosensors. In the case of oxidases, molecules of an enzyme are reduced to their intermediate state and then oxidised by electron acceptors [5,11]. The reduction process as a rule is a specific reaction, while the oxidation of the reduced enzyme by electron acceptors is less specific. Many electron acceptors can be employed in this process [12].

The kinetic analysis of the reaction of a mixture of low and high reactive electron acceptors shows that the reduction of a low reactive electron acceptor may substantially increase if the rate of the cross reaction between these substances is high enough. This phenomenon is employed in biosensors for a high sensitivity determination of synergistic substrates [13–15].

The understanding of the kinetic peculiarities of the biosensors is of crucial importance for their design and optimization. To improve the productivity as well as the efficiency of the biosensor design, a model of the biosensor should be built [16,17]. Starting from the seventies various mathematical models have been widely used as important tools to study and optimize analytical characteristics of actual biosensors [18–22]. A comprehensive reviews on the modelling of the amperometric biosensors have been presented in [23,24]. Biosensors based on synergistic substrates were modelled at steady-state conditions a few years ago [25,26]. Very recently, a laccase-based biosensor utilizing simultaneous substrates conversion was numerically modelled at steady-state as well as transient conditions [27].

The goal of this investigation was to develop a computational model for an effective simulation of amperometric biosensors utilizing the synergistic substrates conversion as well as to investigate the influence of the physical and kinetic parameters on the biosensor response. The developed model is based on non-stationary reaction-diffusion equations [28,29]. The mathematical model comprises three compartments, a layer of enzyme solution entrapped on the electrode surface, a dialysis membrane covering the enzyme layer and an outer diffusion layer. By changing input parameters the output results were numerically analyzed at transition and steady-state conditions. The computational simulation was carried out using the finite difference technique [24,29,30]. The simulation results qualitatively explained and confirmed the experimentally observed effect of the synergistic substrates conversion on the biosensor response [13].

## 2. Mathematical Model

We consider the following kinetic scheme used for the determination of synergistic substrates [13]:

(1a) $$E_{ox}+R\overset{k_{1}}{\to }E_{\text{red}}+P$$

(1b) $$E_{\text{red}}+S_{1}\overset{k_{2}}{\to }E_{ox}+P_{1}$$

(1c) $$E_{\text{red}}+S_{2}\overset{k_{3}}{\to }E_{ox}+P_{2}$$

(1d) $$S_{1}+P_{2}\overset{k_{4}}{\to }P_{1}+S_{2}$$

(1e) $$P_{1}\to S_{1}+n_{1}e^{-}$$

(1f) $$P_{2}\to S_{2}+n_{2}e^{-}$$

where *E_ox_* and *E_red_* stand for oxidized and reduced enzyme forms, respectively, *R* is the enzyme reducer, *S*_1_ and *S*_2_ are the substrates, *P, P*_1_ and *P*_2_ are the products of the reactions, *k*_1_, *k*_2_, *k*_3_, *k*_4_ are the reaction rate constants, and *n*_1_, *n*_2_ are the numbers of electrons involved in a charge transfer at the electrode surface in reactions (1e) and (1f), respectively.

The reactions (1a) and (1b) may be considered as the reduction of a low reactive electron acceptor *S*_1_ by *R* and catalyzed by the enzyme. In the reaction (1a), the enzyme is reduced by the reducer *R*, and an intermediate form of enzyme (*E_red_*) is formed. In the reaction (1b), the electron acceptor *S*_1_ is reduced by the *E_red_*, the product *P*_1_ is formed and enzyme molecules regain their primary oxidized form *E_ox_*.

When the high reactive electron acceptor *S*_2_ is introduced, two more reactions, (1c) and (1d), start. The reaction (1c) represents the enzyme oxidation by the high reactive electron acceptor *S*_2_. The reaction (1d) is called a cross reaction. In the latter reaction, the substrate *S*_1_ oxidizes the product *P*_2_ and regenerates the substrate *S*_2_. This reaction also produces the product *P*_1_.

Reactions (1e) and (1f) are electrochemical reactions that take place on the biosensor electrode. Both reactions are assumed fast and irreversible due to the high electrode potential.

[Fe(CN)_6_]^3−^ ion may be employed as the low reactive electron acceptor *S*_1_ [13,15]. In this case, [Fe(CN)_6_]^4−^ ion would be the reaction product *P*_1_. Cation radicals of 1-(N,N-dimethylamino)-4-(4-morpholino)benzene, 5,10-dihydro-5,10-dimethylphenazine, phenoxazine-10-propionic acid or phenothiazine-10-propionic acid may be used as high reactive electron acceptors (*S*_2_). The reduced forms of those substances would be considered as the corresponding products (*P*_2_) [13].

During the physical experiment [13], the substrate *S*_2_ was introduced into the bulk solution at the moment when the steady-state response to the substrate *S*_1_ was established. The thickness of the diffusion layer was controlled by the rotating magnetic bar and it was estimated using the rotating disk electrode.

### 2.1. Biosensor Structure

**Figure 1.**
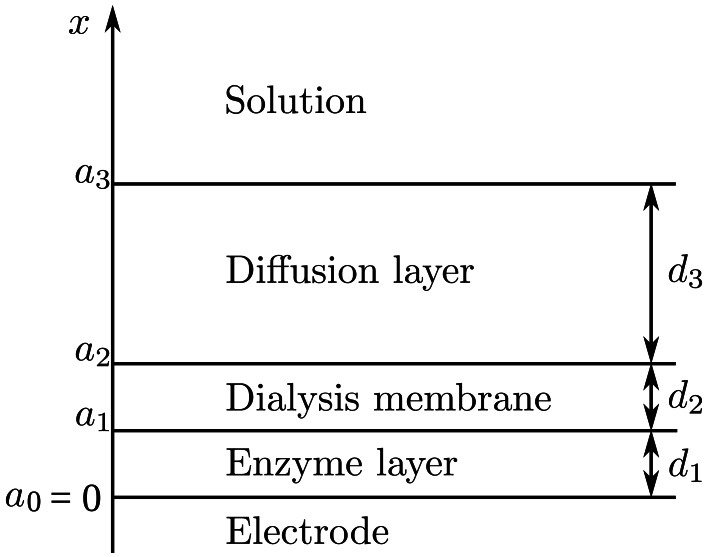
Principal structure of a biosensor.

The biosensor is considered as an electrode with a relatively thin layer of enzyme solution trapped on the surface of the electrode by applying a dialysis membrane [13]. The biosensor model involves four regions: the enzyme layer where the enzymatic and chemical reactions as well as the mass transport by diffusion take place, a dialysis membrane, a diffusion limiting region where only chemical reactions as well as the mass transport by diffusion take place, and a convective region where the analyte concentration is maintained constant, see Figure 1. Assuming a homogeneous distribution of the enzyme in the enzyme layer of the uniform thickness and symmetrical geometry of the dialysis membrane leads to the mathematical model of the biosensor action defined in a one-dimensional-in-space domain [23,24].

Let us define *d*_1_, *d*_2_ and *d*_3_ as the thicknesses of the enzyme, dialysis membrane and diffusion layers, respectively. We will also need values representing the distances between the electrode surface and the boundaries of the regions. Let *a*_1_, *a*_2_ and *a*_3_ be the distances between the electrode surface and one of those boundaries shown in Figure 1.

The diffusion layer (*a*_2_ < *x* < *a*_3_) may be treated as the Nernst diffusion layer [29]. According to the Nernst approach a layer of thickness *d*_3_ remains unchanged with time. It was assumed that away from it the solution is uniform in concentration.

### 2.2. Governing Equations

The governing equations for a chemical reaction network can be formulated by the law of mass action [11,21]. Coupling reactions in the enzyme layer with the one-dimensional-in-space diffusion, described by the Fick's second law, leads to the following equations of the reaction–diffusion type (0 < *x* < *a*_1_, *t* > 0):

(2a) $$\frac{\partial e_{ox}}{\partial t}=-k_{1}e_{ox}r_{1}+k_{2}e_{\text{red}}s_{11}+k_{3}e_{\text{red}}s_{21}$$

(2b) $$\frac{\partial e_{\text{red}}}{\partial t}=k_{1}e_{ox}r_{1}-k_{2}e_{\text{red}}s_{11}-k_{3}e_{\text{red}}s_{21}$$

(2c) $$\frac{\partial r_{1}}{\partial t}=D_{R1}\frac{\partial^{2}r_{1}}{\partial x^{2}}-k_{2}e_{ox}r_{1}$$

(2d) ∂s11∂t=DS11∂s211∂x2−k2ereds11−k4s11p21

(2e) ∂s21∂t=DS21∂s221∂x2−k3ereds21+k4s11p21

(2f) $$\frac{\partial p_{11}}{\partial t}=D_{P_{1}1}\frac{\partial^{2}p_{11}}{\partial x^{2}}+k_{2}e_{\text{red}}s_{11}+k_{4}s_{11}p_{21}$$

(2g) $$\frac{\partial p_{21}}{\partial t}=D_{P_{2}1}\frac{\partial^{2}p_{21}}{\partial x^{2}}+k_{3}e_{\text{red}}s_{21}-k_{4}s_{11}p_{21}$$

where *x* stands for space, *t* is time, *e_ox_*(*x, t*) and *e_red_*(*x, t*) correspond to concentrations of oxidized (*E_ox_*) and reduced (*E_red_*) enzyme, respectively; *s*_11_(*x, t*) and *s*_21_(*x, t*) (*p*_11_(*x, t*) and *p*_21_(*x, t*)) are the concentrations of substrates (products) in the enzyme layer; *r*_1_(*x, t*) is the reducer concentration in the enzyme layer, and *D_R_*_1_, *D_S_*_11_, *D_S_*_21_, *D_P_*_11_, *D_P_*_21_ are the diffusion coefficients of the corresponding substances defined by the subscript. In the definition of the concentrations and diffusion coefficients, here and later in this paper the last numeric subscript label denotes the region of the model, particularly, 1 stands for the enzyme layer. The molecules of both enzyme forms, *E_ox_* and *E_red_*, are considered as immobilized, and therefore there are no diffusion terms in the corresponding equations.

The product *P* does not act as a reactant in any reaction, so its concentration is not used in any further calculations. Therefore, equation system (2) contains no equation for the product *P*.

No enzyme molecules appear in the dialysis membrane as well as in the diffusion layer. Only the reaction (1d) as well as the mass transport by diffusion of the reducer, substrates and products take place in these two regions. The governing equations for both these layers are represented as follows (*a_i_*_−1_ < *x* < *a_i_, t* > 0, *i* = 2, 3):

(3a) $$\frac{\partial r_{i}}{\partial t}=D_{Ri}\frac{\partial^{2}r_{i}}{\partial x^{2}}$$

(3b) $$\frac{\partial s_{1i}}{\partial t}=D_{S_{1}i}\frac{\partial^{2}s_{1i}}{\partial x^{2}}-k_{4}s_{1i}p_{2i}$$

(3c) $$\frac{\partial s_{2i}}{\partial t}=D_{S_{2}i}\frac{\partial^{2}s_{2i}}{\partial x^{2}}+k_{4}s_{1i}p_{2i}$$

(3d) $$\frac{\partial p_{1i}}{\partial t}=D_{P_{1}i}\frac{\partial^{2}p_{1i}}{\partial x^{2}}+k_{4}s_{1i}p_{2i}$$

(3e) $$\frac{\partial p_{2i}}{\partial t}=D_{P2i}\frac{\partial^{2}p_{2i}}{\partial x^{2}}-k_{4}s_{1i}p_{2i}$$

where *i* = 2 corresponds to the dialysis membrane, and *i* = 3 corresponds to the diffusion layer.

### 2.3. Initial Conditions

Let *x* = 0 represents the electrode surface, while *x* = *a*_1_, *x* = *a*_2_ and *x* = *a*_3_ represent the boundaries between the adjacent regions as described in Section 2.1 and shown in Figure 1. The biosensor operation starts when the reducer and the substrates appear in the bulk solution. This is used in the initial conditions (*t* = 0),

(4a) $$\begin{array}{ccc} r_{1}\left(x,0\right)=s_{i1}\left(x,0\right)=p_{i1}\left(x,0\right)=0, & 0\leq x\leq a_{1}, & i=1,2 \end{array}$$

(4b) $$\begin{array}{ccc} e_{\text{red}}\left(x,0\right)=0, & e_{ox}\left(x,0\right)=e_{0}, & 0<x<a_{1} \end{array}$$

(4c) $$\begin{array}{ccc} r_{2}\left(x,0\right)=s_{i2}\left(x,0\right)=p_{i2}\left(x,0\right)=0, & a_{1}\leq x\leq a_{2}, & i=1,2 \end{array}$$

(4d) $$\begin{array}{ccc} p_{i3}\left(x,0\right)=0, & a_{2}\leq x\leq a_{3}, & i=1,2 \end{array}$$

(4e) $$\begin{array}{lll} r_{3}\left(x_{3},0\right)=s_{i3}\left(x,0\right)=0, & a_{2}\leq x<a_{3}, & i=1,2 \end{array}$$

(4f) $$\begin{array}{ll} r_{3}\left(a_{3},0\right)=r_{0}, & s_{13}\left(a_{3},0\right)=s_{10} \end{array}$$

(4g) $$s_{23}\left(a_{3},0\right)=\{\begin{array}{cc} 0, & T_{s_{2}}>0\\ s_{20} & T_{s_{2}}=0 \end{array}$$

where *e*_0_ stands for the total concentration of the enzyme in the enzyme layer (*e*_0_ = *e_ox_*(*x, t*) + *e_red_*(*x, t*), ∀*x, t : x* ε (0, *a*_1_), *t* > 0), *r*_0_ is the reducer concentration in the bulk solution, *s*_10_ and *s*_20_ stand for substrate concentrations in the bulk solution, *T_S_*_2_ is the moment of the substrate *S*_2_ insertion into the bulk solution.

### 2.4. Matching Conditions

On the boundary between two regions having different diffusivities, matching conditions have to be defined (*t* > 0, *i* = 1, 2, *m* = 1, 2),

(5a) $$\begin{array}{cc} {D_{Rm}\frac{\partial r_{m}}{\partial x}|}_{x=a_{m}}={D_{R,m+1}\frac{\partial r_{m+1}}{\partial x}|}_{x=a_{m}}, & r_{m}\left(a_{m},t\right)=r_{m+1}\left(a_{m},t\right) \end{array}$$

(5b) $$\begin{array}{cc} {D_{S_{i}m}\frac{\partial s_{im}}{\partial x}|}_{x=a_{m}}={D_{S_{i},m+1}\frac{\partial s_{i,m+1}}{\partial x}|}_{x=a_{m}}, & s_{im}\left(a_{m},t\right)=s_{i,m+1}\left(a_{m},t\right) \end{array}$$

(5c) $$\begin{array}{cc} {D_{P_{i}m}\frac{\partial p_{im}}{\partial x}|}_{x=a_{m}}={D_{P_{i},m+1}\frac{\partial p_{i,m+1}}{\partial x}|}_{x=a_{m}}, & p_{im}\left(a_{m},t\right)=p_{i,m+1}\left(a_{m},t\right) \end{array}$$

where *m* = 1 corresponds to the boundary between the enzyme layer and the dialysis membrane, whereas *m* = 2 corresponds to the boundary between the dialysis membrane and the diffusion layer.

These conditions mean that fluxes of the reducer, substrates and products through one region are equal to the corresponding fluxes entering the surface of the neighboring region. Concentrations of the reducer, substrates and products in one region versus the neighboring region are assumed to be equal.

### 2.5. Boundary Conditions

In the bulk solution, concentrations of the reducer, substrates and products remain constant (*t* > 0),

(6a) $$r_{3}\left(a_{3},t\right)=r_{0}$$

(6b) $$s_{13}\left(a_{3},t\right)=s_{10}$$

(6c) $$s_{23}\left(a_{3},t\right)=\{\begin{array}{ll} 0, & t<T_{S_{2}}\\ s_{20}, & t\geq T_{S_{2}} \end{array}$$

(6d) $$\begin{array}{ll} p_{i3}\left(a_{3},t\right)=0, & i=1,2 \end{array}$$

The reaction products *P*_1_ and *P*_2_ take part in the electrochemical reactions (1e) and (1f), respectively, at the electrode surface (*x* = 0). Rates of those reactions are so high that the concentrations of *P*_1_ and *P*_2_ at the electrode surface are permanently reduced to zero (*t* > 0) [13],

(7a) $$p_{11}\left(0,t\right)=0$$

(7b) $$p_{21}\left(0,t\right)=0$$

Since the reaction (1e) produces as much *S*_1_ as it consumes *P*_1_, the concentration flux of *P*_1_ on the electrode surface is equal to the concentration flux of *S*_1_ but in opposite direction. The same is also assumed for the reaction (1f) and substances *S*_2_ and *P*_2_. These relations are expressed by the following boundary conditions (*t* > 0):

(8a) $${D_{P_{1}1}\frac{\partial p_{11}}{\partial x}|}_{x=0}={-D_{S_{1}1}\frac{\partial s_{11}}{\partial x}|}_{x=0}$$

(8b) $${D_{P_{2}1}\frac{\partial p_{21}}{\partial x}|}_{x=0}={-D_{S_{2}1}\frac{\partial s_{21}}{\partial x}|}_{x=0}$$

The reducer *R* is electrode-inactive substance, thus its concentration flux on the electrode surface is equal to zero (*t* > 0),

(9) $${D_{R1}\frac{\partial r_{1}}{\partial x}|}_{x=0}=0$$

### 2.6. Biosensor Response

The measured current is usually assumed as the response of an amperometric biosensor in physical experiments. When modelling the biosensor action, due to the direct proportionality of the current to the area of the electrode surface, the current is often normalized with that area [23,31]. The biosensor current density *j*(*t*) at time *t* was expressed explicitly from Faraday's and Fick's laws,

(10a) $${j_{1}\left(t\right)=n_{1}FD_{P_{1}1}\frac{\partial p_{11}}{\partial x}|}_{x=0}$$

(10b) $${j_{2}\left(t\right)=n_{2}FD_{P_{2}1}\frac{\partial p_{21}}{\partial x}|}_{x=0}$$

(10c) $$j\left(t\right)=j_{1}\left(t\right)+j_{2}\left(t\right)$$

where *j*_1_(*t*) and *j*_2_(*t*) are the faradaic current densities generated by the electrochemical reactions (1e) and (1f), respectively, *F* is the Faraday constant, *F* = 96, 486 C/mol.

We assume that the system approaches a steady state as *t* → ∞,

(11a) $$j_{st}=\underset{t\to \infty }{\lim}j\left(t\right)$$

(11b) $$j_{1st}=\underset{t\to \infty }{\lim}j_{1}\left(t\right)$$

(11c) $$j_{2st}=\underset{t\to \infty }{\lim}j_{2}\left(t\right)$$

where *j_st_, j*_1_*_st_* and *j*_2_*_st_* are the steady-state biosensor current densities.

Since the current is generated due to two electrochemical reactions (1e) and (1f), it is important to investigate the influence of each of them to the overall biosensor response. The contribution of the electrochemical reaction (1e) into the overall biosensor response was expressed as the ratio of the density of the steady-state current *j*_1_*_st_* to the density *j_st_* of the overall steady-state current,

(12) $$J_{1}=\frac{j_{1st}}{j_{st}}$$

The sensitivity is also one of the most important characteristics of biosensors [1,2]. The biosensor sensitivity is usually expressed as the gradient of the biosensor current with respect to the concentration of the substrate in the bulk. Since the biosensor current as well as the substrate concentration varies even in orders of magnitude, a dimensionless expression of the sensitivity is preferable [31,32].

The biosensor based on synergistic reactions is designed to measure concentration of the substrate *S*_2_ [13]. The dimensionless biosensor sensitivity *B* to the concentration of the substrate *S*_2_ was defined as follows:

(13) $$B\left(s_{20}\right)=\frac{\text{d}j_{st}\left(s_{20}\right)}{\text{d}s_{20}}\times \frac{s_{20}}{j_{st}\left(s_{20}\right)}$$

where *j_st_*(*s*_20_) is the density of the steady-state biosensor current calculated at the concentration *s*_20_ of the substrate *S*_2_ in the bulk. *B*(*s*_20_) denotes the biosensor sensitivity at that concentration.

The chemical signal amplification caused by a synergistic effect is another important characteristic of this type of biosensors [13]. A measure *Amp* of the synergistic effect was expressed as the ratio of the biosensor response to a substrate *S*_2_-containing analyte to the response to the corresponding *S*_2_-free analyte,

(14) $$\text{Amp}\left(s_{20}\right)=\frac{j_{st}\left(s_{20}\right)}{j_{st}\left(0\right)}$$

where *Amp*(*s*_20_) is the dimensionless ratio of the signal amplification obtained by the insertion of the substrate *S*_2_ of the concentration *s*_20_ into the buffer solution [13,22].

## 3. Numerical Simulation of Biosensor Action

### 3.1. Simulating the Biosensor Operation

Analytical solutions are not usually possible when solving non-linear partial differential equations [19,23,29]. Therefore, the biosensor action was simulated numerically. The simulation was carried out using the finite difference technique [29,30]. An explicit finite difference scheme was built on a uniform discrete grid with 200 points in space direction for each modelled layer corresponding to a certain time moment. The simulator has been programmed by authors in C programming language [33].

In the numerical simulation, the biosensor response time was assumed as the time when the change of the biosensor current remains very small during a relatively long term. A special dimensionless decay rate *ε* was used,

(15) $$\begin{array}{ll} t_{r}\underset{j\left(t\right)>0}{\min}\left\{t:\frac{t}{j\left(t\right)}\left|\frac{\text{d}j\left(t\right)}{\text{d}t}\right|<ɛ\right\}, & j\left(t_{r}\right)\approx j_{st} \end{array}$$

where *t_r_* is the biosensor response time. The decay rate value *ε* = 10^−3^ was used in calculations.

The following values of the model parameters were constant in all numerical simulations, unless stated otherwise [13]:

$$\begin{array}{cc} k_{1} & =1.25\times 10^{4}{\text{M}}^{-1}{\text{s}}^{-1},k_{2}=1.2\times 10^{2}{\text{M}}^{-1}{\text{s}}^{-1}\\ k_{3} & =1.4\times 10^{6}{\text{M}}^{-1}{\text{s}}^{-1},k_{4}=1.4\times 10^{6}{\text{M}}^{-1}{\text{s}}^{-1}\\ e_{0} & =0.04\text{mM},s_{10}=8\text{mM},r_{0}=40\text{mM}\\ D_{R1} & =D_{S_{i}1}=D_{P_{i}1}=3.15\times 10^{-6}{\text{cm}}^{2}/\text{s},i=1,2\\ D_{R2} & =D_{S_{i}2}=D_{P_{i}2}=4.2\times 10^{-7}{\text{cm}}^{2}/\text{s},i=1,2\\ D_{R3} & =D_{S_{i}3}=D_{P_{i}3}=6.3\times 10^{-6}{\text{cm}}^{2}/\text{s},i=1,2\\ d_{1} & =23.3\mu \text{m},d_{2}=18.6\mu \text{m}\\ n_{1} & =n_{2}=1 \end{array}$$

### 3.2. Experimental Validation

**Figure 2.**
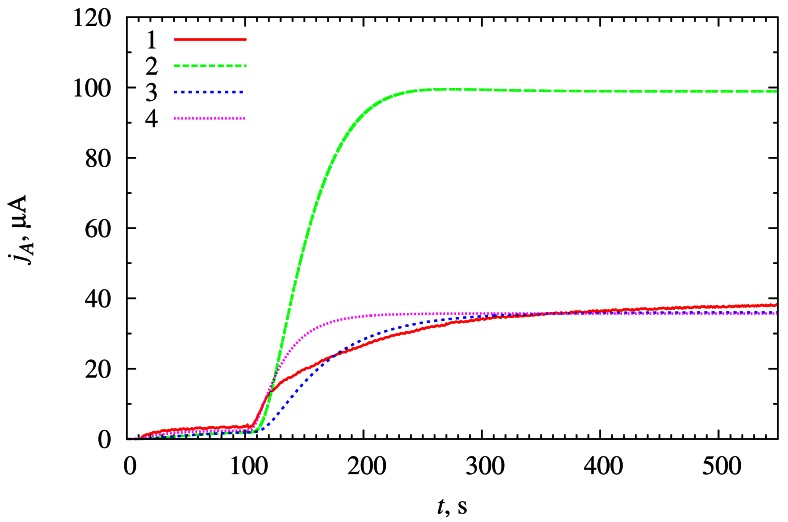
Model validation with experimental data; (1) experimental data, (2)–(4) simulation results. Simulation was carried out at two reaction rate constants (*k*_3_) of the reaction (1c) (1.4 × 10^6^ M^−1^ s^−1^ (2) and 0.35 × 10^6^ M^−1^ s^−1^ (3, 4)) and at two thicknesses (*d*_3_) of the diffusion layer (223 μm (2, 3) and 116.5 μm (4)); *s*_20_ = 39 μM.

The numerical solution of the model (2)–(10) was compared with the experimental data of the research conducted by one of the authors [13]. The results are depicted in Figure 2. The experimental data were shifted by −30 s in time axis and by −1.9 μA in current axis [13]. The correction in time axis was applied in order to coincide the addition of reducer *R* (glucose in this particular case) into the bulk solution at the moment *t* = 0. The correction in current axis was applied in order to eliminate the background current influence to the overall biosensor response.

During physical experiment, the substrate *S*_2_ was added into the bulk solution at the moment *t* = 130 s which corresponds to *t* = 100 s in the simulations. Thus *T_S_*_2_ = 100 s value was used in the numerical simulations. Having simulated density j of the biosensor current, the biosensor current *j_A_* was calculated by multiplying the density by the area *A* of the electrode surface,

(16) $$j_{A}=jA$$

In the physical experiment, the electrode with the surface area of *A* = 0.3 cm^2^ was used [13].

The curve 2 in Figure 2 corresponds to the simulation carried out at the parameters equal to the parameters of the physical experiment [13]. The relative difference between the numerical solution and the experiment data at the moment *T_S_*_2_ (before insertion of *S*_2_) is about 14%. This indicates that mathematical model quite accurately reflects the physical experiment. However, after the insertion of *S*_2_, the accuracy deteriorates. The simulated steady-state current is about 2.5 times higher than the experimental one (Figure 2). This indicates that experiment is really more complex than as defined by the mathematical model. Presumably, the reaction of the reduced enzyme with oxygen, which was unforeseen in the mathematical modelling, is a main factor determining this difference [13]. The difference may be also caused by the limited stability of oxidized heterocyclic compounds and some external diffusion limitation of substrates [13].

The curves 3 and 4 of Figure 2 demonstrate that simulation results may be fitted with the experimental ones by varying the value of the reaction rate constant *k*_3_ and the diffusion layer thickness *d*_3_. However the measured values of the parameters will be used in this paper in further numerical simulations.

Despite this inadequacy with the experiment, the model (2)–(10) seems to be suitable for investigating the kinetic peculiarities and optimizing the configuration of biosensors utilizing the synergistic effect of substrates.

### 3.3. Simulating the Synergistic Effect

The understanding the nature of the synergistic effect is of crucial importance for preparation of highly sensitive biosensors [13,15,26]. For a kinetic analysis of the biosensor operation, let us introduce *&upsilon;̄*_1_*, &upsilon;̄*_2_*, &upsilon;̄*_3_*, &upsilon;̄*_4_ as the average rates of the reactions (1a), (1b), (1c), (1d) taking place in the enzyme layer,

(17a) $${\bar{\upsilon }}_{1}=k_{1}{\bar{e}}_{ox}\bar{r}$$

(17b) $${\bar{\upsilon }}_{2}=k_{2}{\bar{e}}_{\text{red}}{\bar{s}}_{1}$$

(17c) $${\bar{\upsilon }}_{3}=k_{3}{\bar{e}}_{\text{red}}{\bar{s}}_{2}$$

(17d) $${\bar{\upsilon }}_{4}=k_{4}{\bar{s}}_{1}{\bar{p}}_{2}$$

where *r̄, s̄*_1_, s*¯*_2_, *p̄*_1_, *p̄*_2_, *ē_ox_* and *ē_red_* stand for the average concentrations of *R, S*_1_, *S*_2_, *P*_1_, *P*_2_, *E_ox_* and *E_red_* in the enzyme layer.

**Figure 3.**
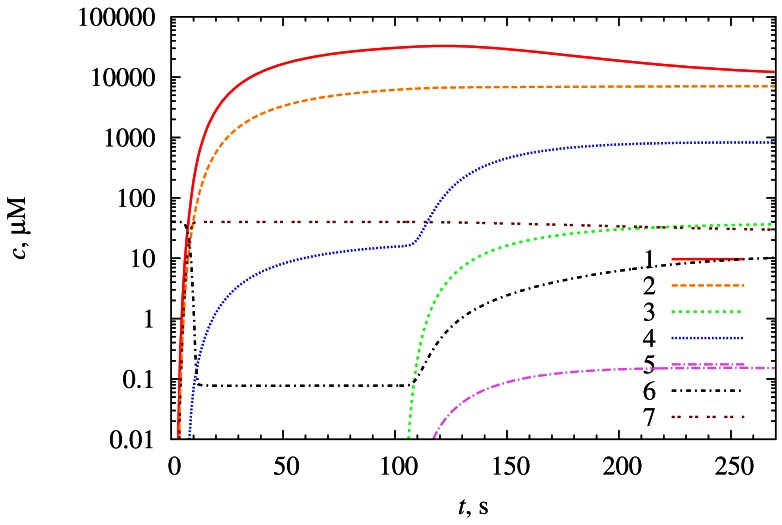
Average concentrations *r̄* (1), *s̄*_1_ (2), s*¯*_2_ (3), *p̄*_1_ (4), *p̄*_2_ (5), *ē_ox_* (6) and *ē_red_* (7).

**Figure 4.**
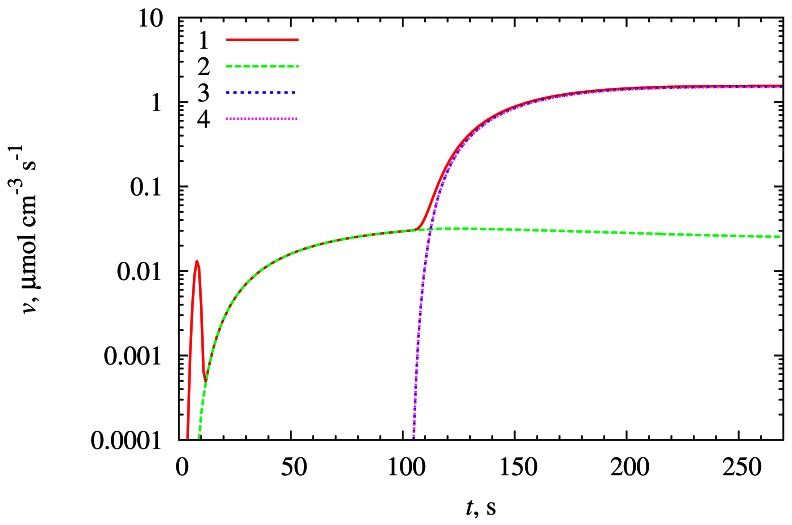
Average rates *&upsilon;̄*_1_ (1), *&upsilon;̄*_2_ (2), *&upsilon;̄*_3_ (3), *&upsilon;̄*_4_ (4) of the reactions (1a)–(1d) taking place in the enzyme layer (curves 3 and 4 coincide).

Figures 3 and 4 show how the synergistic effect occurs. The biosensor parameters were kept the same as in Figure 2.

When *S*_2_ is introduced into the bulk solution, a fast reaction (1c) starts, and a large amount of *E_ox_* and *P*_2_ molecules are produced (see Figure 3). In the meantime the increased concentration of *E_ox_* initiates an increase in the rate of the reaction (1a), molecules of *P*_2_ start to react with *S*_1_ in reaction (1d) (see Figure 4). A decrease of the reducer *R* concentration is the second indication of the increase in the reaction (1a) rate as the reaction consumes more reactant molecules (see Figure 3). The reactions (1a) and (1c) are very strongly interrelated because *E_ox_* is the product of reaction (1c), and at the same time it is the reactant of the reaction (1a) and vice versa with *E_red_* molecule. Similar situation appears with the reactions (1c) and (1d) (molecules of *P*_2_ and *S*_2_).

The rate of the reaction (1c) is controlled by the concentration of *S*_2_. Reactions (1a) and (1d) are accelerated by the reaction (1c) and their rates tend to reach the rate of the reaction (1c). Hence the rates of reactions (1a), (1c) and (1d) are approximately equal during almost all the time of biosensor operation as seen in Figure 4. The rate of the reaction (1b) remains almost unaffected.

The reactions (1b) and (1d) produce the product *P*_1_ which is responsible for the biosensor current. Since the rate of the reaction (1b) remains almost unchanged and the rate of the reaction (1d) is greater than the rate of reaction (1b) by two orders of magnitude, the biosensor response is also increased by two orders of magnitude. In other words, the signal is amplified chemically. This is the desirable outcome of synergistic effect in this type of biosensors [13].

If the synergistic effect would not occur during the biosensor operation, that would mean that biosensor signal is not chemically amplified. A biosensor with no chemical amplification could be too weak for reliable measurement. That could mean the narrower range of biosensor application because biosensor would only work at relatively high concentrations of substrate. So, it is important to choose the correct biosensor parameters that support the occurrence of the synergistic effect.

The law of mass conservation was applied for an additional validation of the numerical solution of the initial boundary value problem Equations (2)–(9). Since no mass transport of the enzyme takes place in the enzyme layer, the equality *e_ox_*(*x, t*) + *e_red_*(*x, t*) = *e*_0_ holds for ∀*x ε* [0, *a*_1_] and ∀*t* ≥ 0. At the steady-state conditions when *∂s_im_/∂t= ∂p_im_/∂t* = 0, the equality *s_im_*(*x, t)* + p*_im_*(*x, t*) = *s*_10_ holds for ∀x ε [*a*_m−1_, *a_m_*] and *t →* ∞, *i* = 1, 2, *m* = 1,2,3. A fulfillment of these conditions can be observed in Figure 3 (please note that concentrations are provided in a logarithmic scale).

### 3.4. Dynamics of Biosensor Current

**Figure 5.**
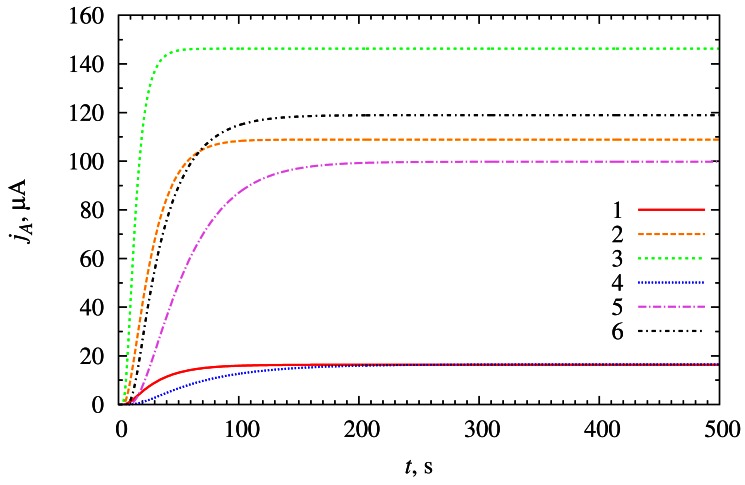
The evolution of the biosensor current *j_A_* at different thicknesses (*d*_3_) of the diffusion layer (116.5 μm (1, 2, 3) and 233 μm (4, 5, 6)) and concentrations (s_20_) of the substrate *S*_2_ (4 μM (1, 4), 40 μM (2, 5) and 400 μM (3, 6).

Figure 5 shows the dynamics of the biosensor current *j_A_* simulated at two thicknesses (*d_3_*) of the diffusion layer and three concentrations (s_20_) of the substrate *S*_2_. For simplicity, here and below, the substrate *S*_2_ is introduced in the bulk solution in the beginning of the experiments (*T_S2_* = 0 s).

At low substrate concentration (s_20_ = 4 μM) the steady-state current of the biosensor is not influenced by the thickness *d*_3_ of the diffusion layer. However at this concentration, a change in the thickness of the diffusion layer slightly changes the curvature of “current *vs.* time” curve and the biosensor with thinner diffusion layer reaches the steady state earlier. This is also correct in cases of higher substrate concentrations (*s*_20_ = 40 μM, *s*_20_ = 400 μM).

From the curves depicted in Figure 5 one can also observe another very important relationship: the steady-state current directly depends on the concentration *s*_20_ of the substrate *S*_2_ in the bulk solution. At higher substrate concentrations (*s*_20_ = 40 μM, s_20_ = 400 μM) it is also observable that steady-state current depends inversely on the thickness *d*_3_ of the diffusion layer. Intensity of the solution stirring plays important role when working with solutions of high concentration of the substrate.

## 4. Results and Discussion

### 4.1. Biosensor Response vs. Substrates Concentrations

**Figure 6.**
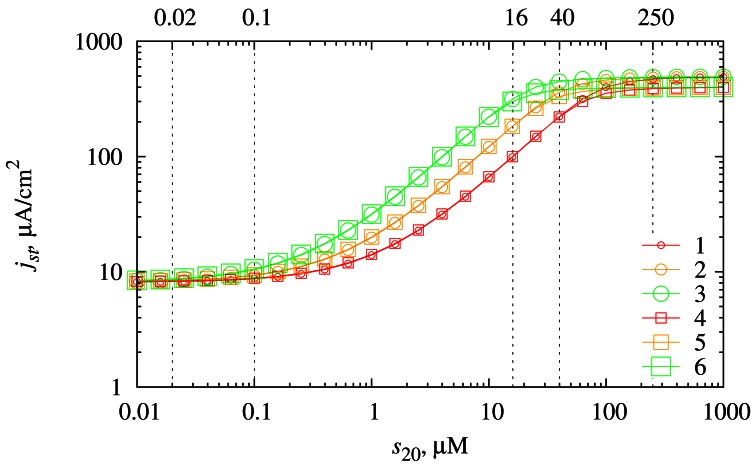
The dependence of the steady-state current density *j_st_* on the substrate concentration *s*_20_ at two thicknesses (*d*_3_) of the diffusion layer (116.5 μm (1, 2, 3) and 233 μm (4, 5, 6)) and three reaction rate constants (*k*_3_) of the reaction (1c) (7 × 10^5^ M^−1^ s^−1^ (1, 4), 1.4 × 10^6^ M^−1^ s^−1^ (2, 5) and 2.8 × 10^6^ M^−1^ s^−1^ (3, 6)).

The dependence of the steady-state biosensor current on the concentration *s*_20_ of the substrate *S*_2_ was investigated at different diffusion layer thicknesses *d*_3_ as well as at different values of the reaction rate constant *k*_3_. The results of the numerical simulations are depicted in Figure 6.

The biosensor may reliably measure the concentration of substrate *S*_2_ at the range of concentrations where the biosensor response is dependent on the concentration *s*_20_ but not dependent on the diffusion layer thickness *d*_3_. As one can observe from Figure 6 this range depends on the reaction rate constant *k*_3_. The lower boundary of this range varies from *s*_20_ ≈ 0.02 μM when *k*_3_ is relatively high (curves 3, 6) to *s*_20_ ≈ 0.1 μM when *k*_3_ is relatively low (curves 1, 4). The upper boundary of this range varies from *s*_20_ ≈ 16 μM when *k*_3_ is relatively high (curves 3, 6) to *s*_20_ ≈ 40 μM when *k*_3_ is relatively low (curves 1, 4). At the concentrations *s*_20_ ≳ 250 μM the biosensor response is not influenced by the concentration of the substrate *S*_2_.

**Figure 7.**
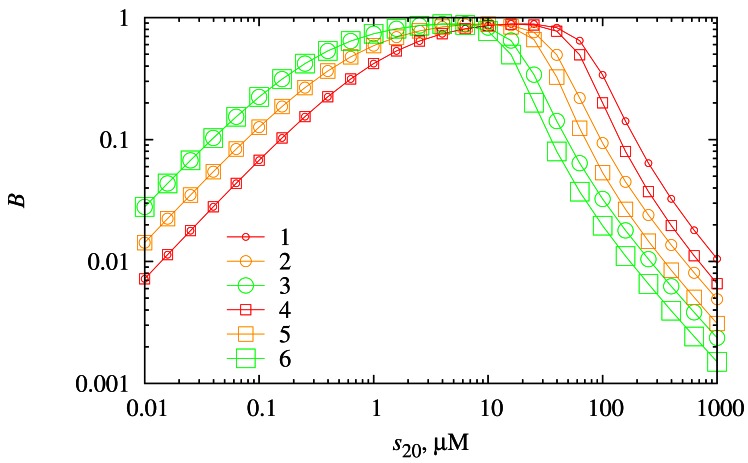
The dependence of the biosensor sensitivity *B* on the substrate concentration *s*_20_. Values of all parameters are the same as in Figure 6.

To investigate the dependence of the biosensor sensitivity on the substrate concentration, the biosensor response was simulated at the same values of the diffusion layer thickness *d*_3_ and the reaction rate constant *k*_3_ as in Figure 6. The calculation results are depicted in Figure 7.

As one can observe from Figure 7, the biosensor shows the highest values of the sensitivity when the concentration of the substrate *S*_2_ is moderate (1 μM ≤ *s*_20_ ≤ 10 μM). At low substrate *S*_2_ concentrations (0.01 μM ≤ *s*_20_ < 1 μM) as well as at high concentrations of *S*_2_ (10 μM < *s*_20_ ≤ 1,000 μM) biosensor exhibits poor sensitivity.

At low substrate concentrations, a higher *k*_3_ yields a higher biosensor sensitivity, whereas the thickness *d*_3_ of the diffusion layer does not influence the biosensor response at this concentration range. At high substrate concentrations, *k*_3_ has the opposite effect on the biosensor sensitivity compared with a low substrate concentration range. A higher *k*_3_ yields a lower biosensor sensitivity. At this concentration range, the thickness *d*_3_ of the diffusion layer does influence the biosensor sensitivity. A thicker diffusion layer diminishes the value of biosensor sensitivity.

**Figure 8.**
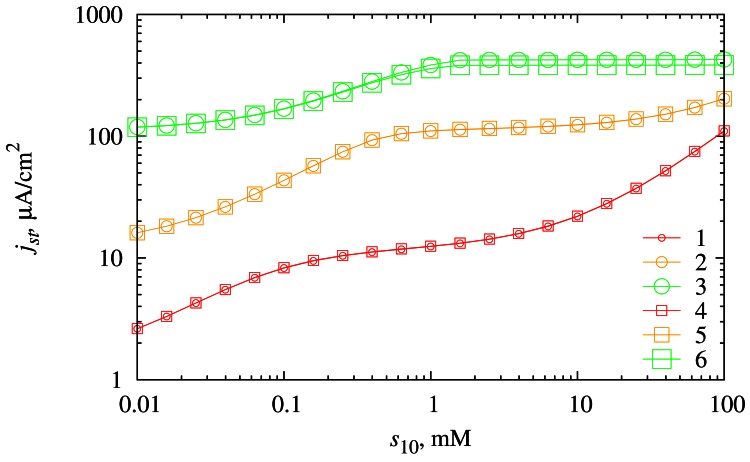
The dependence of the steady-state current density *j*_st_ on the substrate concentration *s*_10_ at two thicknesses (*d*_3_) of the diffusion layer (116.5 μm (1, 2, 3) and 233 μm (4, 5, 6)) and three concentrations (*s*_20_) of the substrate *S*_2_ (1 μM (1, 4), 10 μM (2, 5) and 100 μM (3, 6)).

Although this type biosensors are designed to measure the concentration *s*_20_ of the substrate *S*_2_*,* it is also important to investigate the biosensor response dependency on the concentration *s*_10_ of the substrate *S*_1_ in order to optimize the biosensors. The dependence of the steady-state biosensor current *j_st_* on the concentration *s*_10_ was investigated at different values of diffusion layer thickness *d*_3_ as well as at different concentrations (*s*_20_) of the substrate *S*_2_. The simulation results are depicted in Figure 8.

As we can observe from the curves in Figure 8, the biosensor response directly depends on the concentration *s*_10_. In cases of low and moderate substrate concentrations of *S*_2_, this dependency holds through the whole range of investigated *s*_10_ concentrations. At high concentrations of *S*_2_ (*s*_20_ = 100 μM), this dependency is valid only for low concentrations of *S*_1_ (*s*_10_ < 1mM). At high concentrations of *S*_1_ (*s*_10_ > 1 mM) the biosensor response becomes practically insensitive to the change of *s*_10_.

The thickness *d*_3_ of the diffusion layer has no noticeable influence on the biosensor response at the majority of the biosensor parameters investigated. Only at high concentrations of *S*_1_ and high concentrations of *S*_2_ the effect of the diffusion layer thickness becomes noticeable.

### 4.2. The Composition of Biosensor Response

The biosensor response is determined by two electrochemical reactions (1e) and (1f). It is important to understand the role of each process at certain values of the biosensor parameters.

**Figure 9.**
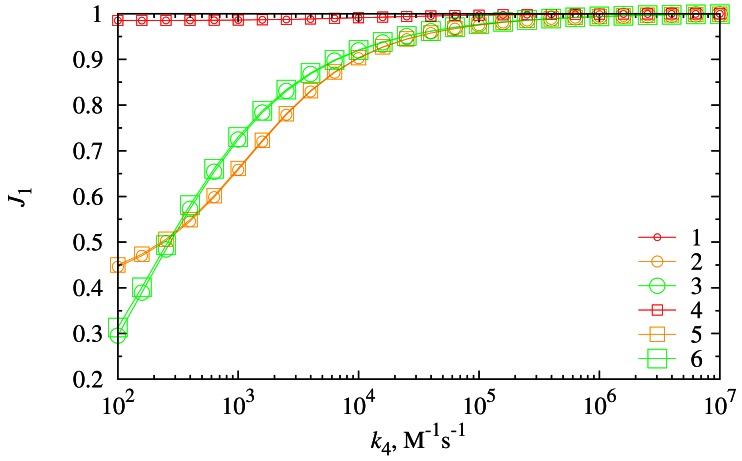
The dependence of the ratio *J*_1_on the reaction rate *k*_4_ at two thicknesses (*d*_3_) of the diffusion layer (116.5 μm (1, 2, 3) and 233 μm (4, 5, 6)) and three concentrations (*s*_20_) of the substrate *S*_2_ (0.1 μM (1, 4), 10 μM (2, 5) and 1, 000 μM (3, 6)).

The dependence of the biosensor response composition on the reaction rate constant *k*_4_ was investigated at different diffusion layer thicknesses (*d*_3_) as well as at different values of the substrate concentration *s*_20_. Results of the numerical simulation are depicted in Figure 9.

As one can see in Figure 9, the ratio *J*_1_ of the density *j*_1_*_st_* of the steady-state current generated by the electrochemical reactions (1e) to the density *j_st_* of the overall steady-state current directly depends on the reaction rate constant *k*_4_. This can be explained by the reaction (1d). The faster the reaction (1d) the more *P*_2_ consumes, and molecules of *P*_2_ do not reach electrode surface.

At low *s*_20_ concentrations, a value of *k*_4_ only slightly influences the composition of the biosensor response (curves 1 and 4). The biosensor response is almost entirely generated by the faradaic process (1e) at such concentrations of *S*_2_. As one can see from the reaction scheme (1), a low concentration of *S*_2_ directly cause a low concentration of *P*_2_. However at a high concentration *s*_20_ of *S*_2_, a value of the reaction rate constant *k*_4_ makes a major impact on the composition of the biosensor response. When *k*_4_ is small enough, the ratio *J*_1_ is less than 0.5, *i.e*., the main part of the biosensor response is generated by the faradaic process (1f).

Figure 9 also shows that the thickness *d*_3_ of the diffusion layer only slightly influences the composition of the biosensor response.

### 4.3. The Role of Substrates Synergistic Conversion in the Biosensor Response

**Figure 10.**
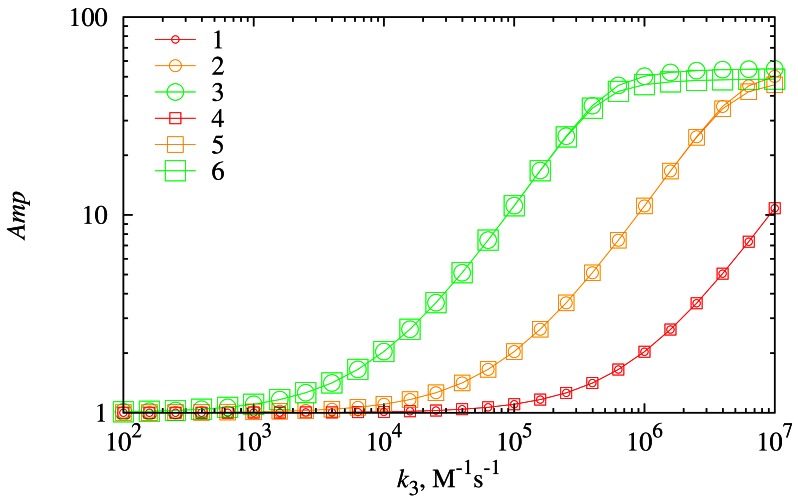
The dependence of the chemical amplification *Amp* on the reaction rate constant *k*_3_ at two thicknesses (*d*_3_) of the diffusion layer (116.5 μm (1, 2, 3) and 233 μm (4, 5, 6)) and three concentrations (*s*_20_) of *S*_2_ (1 μM (1, 4), 10 μM (2, 5) and 100 μM (3, 6)).

The synergistic effect was analyzed with respect to the chemical amplification *Amp*. The dependence of the chemical amplification on the reaction rate constant *k*_3_ was investigated at different diffusion layer thicknesses (*d*_3_) as well as at different values of the substrate concentration *s*_20_. Results of the numerical simulation are depicted in Figure 10.

The direct influence of the reaction rate constant *k*_3_ on the *Amp* is the main dependency that can be observed in Figure 10. At a higher rate constant (*k*_3_) of the biochemical reaction (1c) more molecules of *P*_2_ are produced. Because of this, the rate of the reaction (1d) increases, and eventually the amount of *P*_1_ also increases. Product *P*_1_ is the main generator of the current as was shown in the Section 4.2.

As one can also see in Figure 10, a higher concentration *s*_20_ of *S*_2_ corresponds to a higher chemical amplification *Amp*. At low values of *k*_3_ (*k*_3_ < 10^3^ M^−1^ s^−1^) this effect is rather weak, whereas at moderate values of *k*_3_ (from 10^4^ to 10^6^ M^−1^ s^−1^) this effect is strongly expressed. However, at high values of the reaction rate constant *k*_3_ (*k*_3_ > 10^7^ M^−1^ s^−1^) this effect noticeably diminishes again. A positive influence of *s*_20_ on the amplification can be explained by the increase of the rate of reaction (1c) that is caused by the increase of the concentration of one of the reactants. At low values of *k*_3_ this is not so obvious because the increase in the reactant concentration does not counterweigh low values of *k*_3_. At high values of *k*_3_ the system arrives at the point where the chemical amplification *Amp* is not dependent on the reaction rate constant *k*_3_. The influence of the diffusion layer thickness *d*_3_ appears noticeable only at high values of *k*_3_.

Similar limitations of the rate of the synergistic process have been also observed experimentally using glucose oxidase biosensors utilizing the synergistic substrates conversion [13].

**Figure 11.**
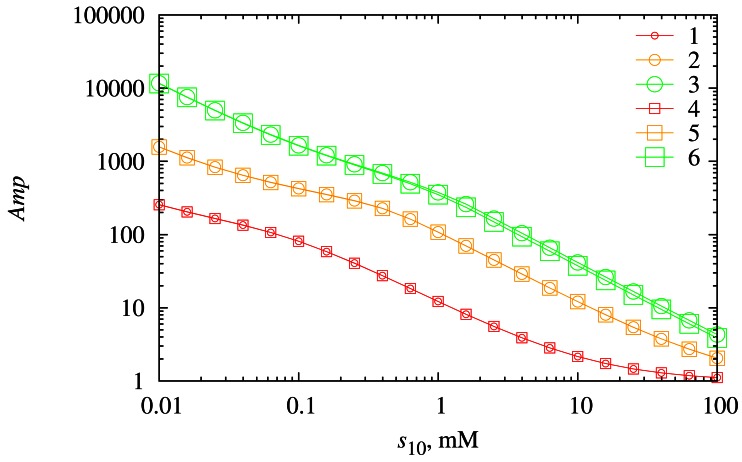
The dependence of the chemical amplification *Amp* on the substrate concentration *s*_10_ at two thicknesses (*d*_3_) of the diffusion layer (116.5 μm (1, 2, 3) and 233 μm (4, 5, 6)) and three concentrations (*s*_20_) of the substrate *S*_2_ (1 μM (1, 4), 10 μM (2, 5) and 100 μM (3, 6)).

The amplification of the biosensor current was also investigated with respect to the substrate *S*_1_ concentration. Numerical experiments were conducted at different diffusion layer thicknesses (*d*_3_) as well as at different concentrations (*s*_20_). The results of the numerical simulation are depicted in Figure 11.

As one can see in Figure 11 the biosensor exhibits the highest values of the chemical signal amplification *Amp* at low concentrations of substrate *S*_1_ and the lowest values of *Amp* at high values of *s*_10_. So, the influence of *s*_10_ on the biosensor response that was observed in Figure 8 is diminished by the chemical amplification, because the magnitude of the amplification buffers the impact of the substrate *S*_1_ concentration change. When the concentration *s*_10_ is low, the response of the biosensor is highly amplified by the synergistic effect and vice versa in the case of high *s*_10_.

The thickness of the diffusion layer only slightly influences the chemical amplification throughout the range of the concentration *s*_10_ that was used in this investigation. A slight influence of the diffusion layer thickness *d*_3_ is observed only in the case of high concentrations of both substrates, *S*_1_ and *S*_2_.

## 5. Conclusions

The developed mathematical model (2)–(10) is suitable for the modelling of an amperometric biosensor with synergistic substrates conversion. The model can be used as a tool for the biosensor optimization prior to the development stage of an actual biosensor.

The investigation of the biosensor response showed that the range of concentrations, where the biosensor reliably operates, depends on the reaction rate constant *k*_3_. In cases when *k*_3_ is lower the biosensor may be used to measure higher concentrations *s*_20_. In cases when *k*_3_ is higher the biosensor may be used to measure lower concentrations *s*_20_ (Figure 6).

The biosensor has the highest sensitivity at moderate concentrations of the substrate *S*_2_ (Figure 7). At low and high concentrations of *S*_2_, the biosensor sensitivity is quite low.

The biosensor response is directly dependent on the concentration *s*_10_ of the substrate *S*_1_. Low and moderate concentrations of *S*_1_ are preferable, because at these concentrations the biosensor response is practically independent from the diffusion layer thickness (Figure 8).

The biosensor response almost entirely depends on the electrochemical reaction (1e) (Figure 9). The electrochemical reaction (1f) may generate a significant current only in cases when the reaction rate constant of cross reaction (1d) is low and the concentration *s*_20_ of *S*_2_ is moderate or high (Figure 9).

The biosensor exhibits the highest values of the chemical amplification in cases when the reaction rate constant *k*_3_ as well as the concentration *s*_20_ are the high (Figure 10). The diffusion has a minor effect on the rate *Amp* of the chemical amplification. A thicker diffusion layer weakens the chemical amplification only at high values of the reaction rate constant *k*_3_ and high concentrations of *S*_2_. The amplification ratio *Amp* inversely depends on the concentration *s*_10_ of the substrate *S*_1_ (Figure 11). This effect is somewhat opposite to the *s*_10_ effect on the biosensor response.

To prove conclusions made the physical experiments are running using glucose dehydrogenase as well as laccase-based biosensors with different configurations. A more precise computational model implying the reaction of the reduced enzyme with oxygen in addition to the reactions (1e) and (1f) is now under development for a more accurate simulation of the biosensor action.
